# Effect of Prostaglandin Analogues on Hyaluronan Production of Orbital Fibroblasts

**DOI:** 10.3390/biomedicines14092128

**Published:** 2026-09-21

**Authors:** Sara Makhoul, Erika Galgoczi, Monika Katko, Janos K. Aranyosi, Noémi Széll, Sandor Horvath, Istvan Orsos, Zsanett Molnar, Mariann Fodor, Endre V. Nagy, Bernadett Ujhelyi

**Affiliations:** 1 Department of Ophthalmology, Faculty of Medicine, University of Debrecen, 4032 Debrecen, Hungary; makhoul.sara@med.unideb.hu (S.M.); ifj.dr.aranyosi.janos@med.unideb.hu (J.K.A.); szell.noemi@med.unideb.hu (N.S.); mfodor@med.unideb.hu (M.F.); bujhelyi@med.unideb.hu (B.U.); 2 Doctoral School of Clinical Medicine, University of Debrecen, 4032 Debrecen, Hungary; 3 Division of Endocrinology, Department of Internal Medicine, Faculty of Medicine, University of Debrecen, 4032 Debrecen, Hungary; galgoczi.erika@med.unideb.hu (E.G.); katko.monika@med.unideb.hu (M.K.); orsosistvan99@gmail.com (I.O.); mzsanett82@gmail.com (Z.M.); 4 Faculty of Medicine, University of Debrecen, 4032 Debrecen, Hungary; sunker2121@gmail.com; 5 Doctoral School of Medical Sciences, University of Debrecen, 4032 Debrecen, Hungary

**Keywords:** prostaglandin analogues, hyaluronan, thyroid eye disease, orbital fibroblasts, PDGF-BB

## Abstract

**Background:** In thyroid eye disease (TED) pathogenesis, a major contributor is hyaluronan (HA) overproduction by orbital fibroblasts (OF). TED patients often receive prostaglandin analogues (PGA) for elevated intraocular pressure. Their direct effects on OFs, however, remain unclear. **Methods:** OFs were established from connective tissues obtained from TED patients (*n* = 4) and non-TED controls (*n* = 4) during orbital surgeries. HA levels in primary OF culture media, and in the OFs’ pericellular coat, were measured by ELISA. Cultures were performed with or without platelet-derived growth factor BB (PDGF-BB) stimulation, and treated for 72 h with 40 µM prostaglandin F2α (PGF2α), latanoprost, bimatoprost or tafluprost. mRNA expressions of HA synthases (HAS1, HAS2, HAS3) and HA-degrading enzymes (HYAL1, HYAL2, CEMIP) were quantified by RT-PCR. Proliferation and metabolic activity were analysed using BrdU incorporation and MTT assays. **Results:** PGF2α and each PGA reduced HA levels in the pericellular coat (*p* < 0.001), while only PGAs reduced HA concentration in the culture medium. Tafluprost and latanoprost suppressed PDGF-BB-stimulated HA synthesis to or below unstimulated levels, respectively (*p* < 0.001). Latanoprost markedly decreased *HAS2* mRNA expression (*p* < 0.001), even under PDGF-BB stimulation (*p* < 0.001). PGF2α and each PGA increased *CEMIP* mRNA expression (*p* < 0.001). Latanoprost decreased fibroblast proliferation and metabolic activity with and without PDGF-BB stimulation (*p* < 0.05). **Conclusions:** These in vitro findings suggest that PGAs modulate HA turnover in orbital fibroblasts through reduced HA synthesis and potentially enhanced degradation. While these observations are confined to cultured cells, they offer a hypothesis for potential clinical relevance in TED, which requires further in vivo validation.

## 1. Introduction

Thyroid eye disease (TED) is a complex autoimmune disorder most commonly associated with Graves’ disease. TED is characterised by the expansion of orbital soft tissues driven by enhanced fibroblast (OF) proliferation, adipocyte differentiation, and increased extracellular matrix (ECM), especially hyaluronan (HA) deposition [1]. Although several details of the pathogenesis of TED are not clear, current evidence indicates that both cellular and humoral immune processes are essential in this respect [2]. T lymphocytes activate OFs via the CD40–CD154 pathway resulting in oedema, fibrosis, adipogenesis and collagen deposition through increased production of inflammatory mediators [3,4]. Important clinical consequences are limited eye movement and proptosis [5]. HA has high water-binding capacity up to 1000 times its own weight [6], and the overproduction of HA results in oedematous tissue swelling [7]. Hyaluronan synthases (HAS), namely HAS1 and HAS2, are responsible for the high molecular weight (HMW) HA, and HAS3 for the medium molecular weight (MMW) HA production [8]. The degradation of HA is mediated by hyaluronidases, HYAL1 and HYAL2 [9], and CEMIP [10]. Inflammation and reactive oxygen species contribute to the accumulation of low molecular weight (LMW) HA [11]. HA size is an important determinant of the biophysical and biochemical properties [12] and physiological roles [13] of HA. In vivo HA molecules are localised in the pericellular coat anchored to synthases or attached to CD44 surface receptors [14].

Elevated intraocular pressure (IOP) in thyroid eye disease (TED) is multifactorial and related to orbital tissue expansion and impaired aqueous humour drainage. Expansion of the orbital connective and adipose tissues and enlargement of the extraocular muscles may increase intraorbital pressure, which might cause mechanical compression of the globe. Orbital congestion increases episcleral venous pressure that might reduce episcleral aqueous humour outflow. In addition, restrictive extraocular myopathy and proptosis may contribute to higher IOP readings, particularly in certain gaze positions. Accordingly, elevated IOP in TED should not be attributed solely to impaired aqueous humour outflow through the uveoscleral pathway, but rather to the combined effects of orbital congestion, extraocular muscle involvement and elevated episcleral venous pressure [15,16,17,18].

In patients with TED, elevated IOP affects 6.8% of cases during the active phase of the disease [16,17]. Increased IOP facilitates the development of secondary open-angle glaucoma (OAG), which is present in around 1.6% of TED patients [16]. Distinguishing between ocular hypertension (OHT) and OAG is crucial, as the presence of OHT does not indicate glaucomatous optic nerve damage [17]. Identifying patients at risk for developing true glaucomatous optic neuropathy is essential to prevent unnecessary or potentially harmful interventions. In OAG, prostaglandin analogues (PGAs) are first-line treatment options, as they effectively decrease IOP through the uveoscleral pathway [18]. Prostaglandins are hormone-like compounds derived from lipids that regulate numerous physiological processes, including vascular tone, platelet function, IOP, inflammatory processes, and cell growth or differentiation [19]. Their side effects may include conjunctival hyperaemia, ocular irritation, increased iris pigmentation, as well as periocular tissue changes, such as excessive eyelash growth (hypertrichosis), increased pigmentation and deepening of the upper eyelid sulcus (DUES) [20,21]. The most commonly used eye drops for glaucoma treatment are those containing latanoprost, travoprost, bimatoprost, and tafluprost, all of which are synthetic analogues of prostaglandin F_2_α (PGF2α) [18].

It has been known that at tissue level, PGAs influence fibroblast proliferation, ECM and collagen production, as well as inflammatory processes [22,23]. Their activity is modulated by the cellular environment, receptor expression, and signalling pathways, which may elicit diverse, often opposing immune responses [23]. The result of the long local administration of PGAs has been linked to DUES [19]; inhibition of adipocyte differentiation and lipid accumulation of preadipocytes may be a potential mechanism of DUES [24,25,26]. The potential role of PGAs in the regulation of hyaluronan (HA) production of OFs, which may be beneficial in TED, has not been studied. We aimed to investigate the effect of PGF2α and its analogues on the HA production of human OFs.

## 2. Materials and Methods

### 2.1. Materials

DMEM high glucose, stable glutamine (SG), foetal bovine serum (FBS) and Dulbecco’s phosphate-buffered saline (DPBS) were purchased from Biosera (Nuaille, France). TrypLE Express was purchased from Gibco (Thermo Fisher Scientific, Waltham, MA, USA). Prostaglandin F2α (PGF2α), prostaglandin analogues (PGAs): latanoprost, tafluprost and bimatoprost, dimethyl sulfoxide (DMSO) and 3-(4,5-dimethylthiazol-2-yl)-2,5 diphenyl tetrazolium bromide (MTT) were purchased from Sigma Aldrich (St. Louis, MO, USA). Recombinant human platelet-derived growth factor BB (PDGF-BB) was purchased from R&D Systems (Bio-Techne, Minneapolis, MN, USA). Bioline Agarose was purchased from Meridian Bioscience (Cincinnati, OH, USA). Proteinase K, Tris, bromophenol blue, sucrose, chloroform, sodium dodecyl sulfate (SDS), Stains All, and Tris–borate–EDTA (TBE) buffer were purchased from Merck KGaA (Darmstadt, Germany). TRI solution was purchased from Molecular Research Center Inc. (Cincinnati, OH, USA). Cell proliferation ELISA BrdU colorimetric kit was purchased from Roche (F. Hoffmann-La Roche Ltd., Basel, Switzerland). A High-Capacity cDNA Reverse Transcription Kit and TaqMan Gene Expression Assays were purchased from Applied Biosystems (Thermo Fisher Scientific, Waltham, MA, USA). Different molecular weight HA standards were purchased from Tocris (Bio-Techne, Minneapolis, MN, USA).

### 2.2. Patients’ Tissue Samples and Cell Cultures

The orbital connective tissue samples were originated from decompression surgery of four TED patients (3 females and 1 male) during the inactive phase of the disease. The mean TED duration from disease onset was 4.75 (min: 1, max: 8) years. The mean age of TED patients was 51 years (min: 37, max: 63). All patients received at least one full course of intravenous glucocorticoid treatment [26]. Two patients underwent orbital irradiation and one patient received pentoxifylline. None of the patients received glucocorticoid treatment or orbital irradiation during the last 12 months prior to surgery. The four control orbital connective tissue explants were obtained from four patients (2 females and 2 males) who underwent enucleation due to malignant choroidal melanoma, with no history of thyroid disease or TED. The mean age of non-TED patients was 61 (min: 38, max: 82) years. Primary OF cultures were established as described by Bahn et al. (1987) [27]. For all experiments, OFs were plated at a standardised density of 1.56 × 10^4^ cells/cm^2^ in 24-well or 96-well plates in DMEM High Glucose supplemented with 10% (*v*/*v*) FBS, penicillin/streptomycin, and stable glutamine and cultured for 72 h. All experiments were performed at least 3 times and carried out in triplicate.

All patients provided written consent. The study was approved by the Regional and Institutional Research Ethics Committee of the University of Debrecen (RKEB/IKEB 5913-2012, and date of approval 27 November 2012).

### 2.3. Preparing Solutions of PGF2α and PGAs for Cell Culture

Of PGF2α and PGAs, 10 mM stock solutions were prepared in DMSO according to the manufacturer’s instructions. Culture medium was added to reach the final tissue culture concentration; dose–response relationships were examined in the range of 1–40 µM. A maximum of 40 µM was used, since higher DMSO end-concentrations caused fibroblast decay.

### 2.4. Cell Metabolic Activity Assay

Cells were plated in 96-well plates at confluent density and cultured for 72 h at 37 °C in a CO_2_ incubator with PGF2α, a PGA, or vehicle as control, with or without PDGF-BB stimulation. Briefly, 10 µL MTT (5 mg/mL) per 100 µL medium was added at the 69th hour of the experiment. The cells were incubated for 3 h in a CO_2_ incubator at 37 °C. The formed blue formazan crystals were dissolved in DMSO. The absorbance was detected at 550 nm (reference wavelength: 660 nm) using Synergy H1 Microplate Reader (Agilent Technologies Inc., Santa Clara, CA, USA). Cell metabolic activity was expressed as optical density compared to that of untreated controls or PDGF-BB-stimulated cells.

### 2.5. Cell Proliferation Assay

The proliferation assay was performed in 96-well plates according to the manufacturer’s instructions. Briefly, the cells from the 70th hour of the experiment were incubated for 2 h with 5-bromo-2′-deoxyuridine (BrdU) solution. Then the media were removed and the cultures were fixed using FixDenat solution. After fixation, peroxidase-conjugated anti-BrdU antibody was added for 90 min. Finally, the substrate was introduced for 10 min, and the reaction was stopped with 2 N H_2_SO_4_. The absorbance at 450 nm (reference wavelength: 620 nm) was detected using a Beckman Coulter, DTX 880 Multimode Detector (Beckman Coulter Inc., Brea, CA, USA). The proliferation potential was expressed as optical density compared to that of untreated controls or PDGF-BB-stimulated cells.

### 2.6. Quantitation of Hyaluronan

For measurement of HA either present in the pericellular coat or secreted into the culture media, the OFs were plated in 96-well plates. For the measurement of the secreted HA, after the 72 h treatment, culture media were removed and stored at −20 °C. For the measurement of the HA content in the pericellular coat, cells were washed twice with PBS, then treated with 0.05% (*w*/*v*) trypsin–EDTA solution at 37 °C for 20 min. FBS (10%) was used to inactivate trypsin. In order to remove any cell debris, the samples were centrifuged at 1000× *g* for 5 min [28,29,30], supernatants were collected, and stored at −20 °C. HA concentrations in both sample types were measured using the DuoSet Hyaluronan ELISA kit (R&D Systems, Minneapolis, MN, USA) according to the manufacturer’s instructions using a DTX 880 Multimode Detector (Beckman Coulter, Brea, CA, USA).

### 2.7. HA Isolation and Separation by Agarose Gel Electrophoresis

The details of the HA isolation technique used were described earlier [30]. Briefly, samples of the culture medium were incubated with Proteinase K solution for 4 h, then mixed with 96% ethanol and stored at −20 °C for overnight. The precipitated HA was separated by centrifugation and, in the last step, was dissolved in TBE buffer. The electrophoresis was performed in 1.5% agarose. After the pre-run of the gel, the prepared HA samples were mixed with of 0.02% bromophenol blue. The run was performed for 0.5 h at constant 20 V and then for 3.5 h at constant 40 V. The gel was stained with Stains All solution (0.005% dye in 50% ethanol) overnight. After destaining, the detection was performed using LED transilluminator and photographed. 

### 2.8. Evaluation of Molecular Weight Distribution

The evaluation process of the gels is described in detail in our previous paper [30]. Briefly, the stained gels were photographed, and the images were evaluated using Image Studio Digits 5.2 (LI-COR Biotechnology, Bad Homburg, Germany) software. To enable statistical comparison, the lane profiles of each sample run on the gel were divided from top to bottom into 23 equal sections based on specified molecular weight hyaluronan standards: HMW-HA (>950 kDa, sections 1–6), MMW-HA (75–1000 kDa, sections 7–19), and LMW-HA (<40 kDa, sections 20–23). Due to the uncertainties arising from the HA standards, the definition of the high, medium, and low molecular weight thresholds is an operational definition based on our analytical judgement, integrating both the gel images and the reference standards. According to the published literature, we classified the 40–75 kDa interval—unresolved by the standards—within the medium-molecular weight fraction. The statistical analyses compared densitometric readings from 4 TED and 4 non-TED OF cultures. Data were visualised using GraphPad Prism 8 (Boston, MA, USA).

### 2.9. Real-Time Polymerase Chain Reactions

The RNA was isolated using TRI reagent. For making the cDNA library, a High-Capacity cDNA Reverse Transcription Kit was used. The TaqMan Gene Expression Assay was used for the detection of the expression of hyaluronan synthases and hyaluronidases (*HAS1*–Hs00987418_m1, *HAS2*–Hs00193435_m1, *HAS3*–Hs00193436, *HYAL1*–Hs00201046_m1, *HYAL2*–Hs01117343_g1, *CEMIP*–Hs01552114_m1). The results were normalised to *GAPDH* (Hs02786624_g1) mRNA levels by the ΔCT method. The reactions were performed by the CFX Opus 96 Real-time PCR System (BioRad, Hercules, CA, USA).

### 2.10. Statistical Analysis

The statistical analyses were performed using STATISTICA 14.1.0 software (TIBCO Software Inc., Palo Alto, CA, USA). Repeated measures ANOVA with treatment as the within-subjects factor and origin of fibroblasts (TED and non-TED) as between-subjects factor followed by Fisher LSD post hoc test was used to evaluate the differences between groups. Individual donors served as the biological experimental units. Technical replicates from repeated experiments for each cell culture derived from different donors were averaged prior to statistical analysis to ensure that technical replicates and repeated experiments were not treated as independent biological observations. Data are presented as mean ± standard error of mean (SEM). Statistical significance was accepted at *p* values < 0.05.

## 3. Results

**Figure 1:**
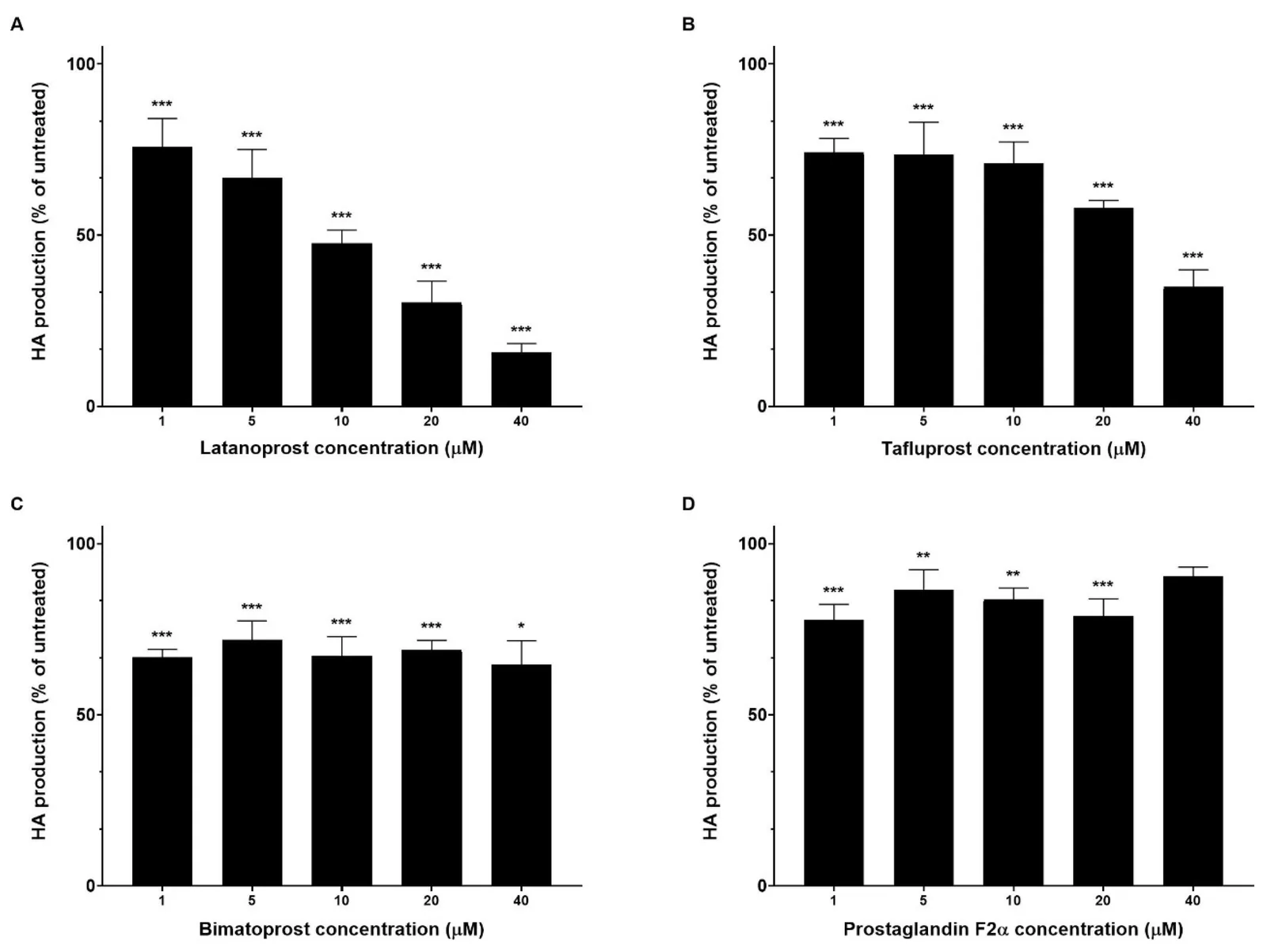
The dose-dependent effect of PGAs and PGF2α on hyaluronan production of orbital fibroblasts in culture media. (**A**) Latanoprost, (**B**) Tafluprost, (**C**) Bimatoprost and (**D**) Prostaglandin F2α (OFs *n* = 4). Data were analysed using repeated measures ANOVA followed by Fisher LSD post hoc test. Results are shown as mean ± SEM. *—results compared to untreated; * *p* < 0.05, ** *p* < 0.01, *** *p* < 0.001.

After 72 h of treatment with different concentration of PGF2α or different PGAs, latanoprost and tafluprost resulted in a dose-dependent decrease in HA levels in the culture media (Figure 1A,B). Compared to untreated cultures, bimatoprost and PGF2α had only a mild reducing effect at all tested concentrations (Figure 1C,D). The strongest decrease in HA production was observed at a concentration of 40 µM for latanoprost and tafluprost (*p* < 0.001 and *p* = 0.0013, respectively); furthermore, the effects of these two PGAs were not statistically different from each other (*p* = 0.248). At this concentration, bimatoprost and PGF2α caused less inhibition compared to latanoprost (*p* = 0.0055 and *p* = 0.0034, respectively). Based on these data, the 40 µM concentration was chosen for further experiments (Figure 1).

**Figure 2:**
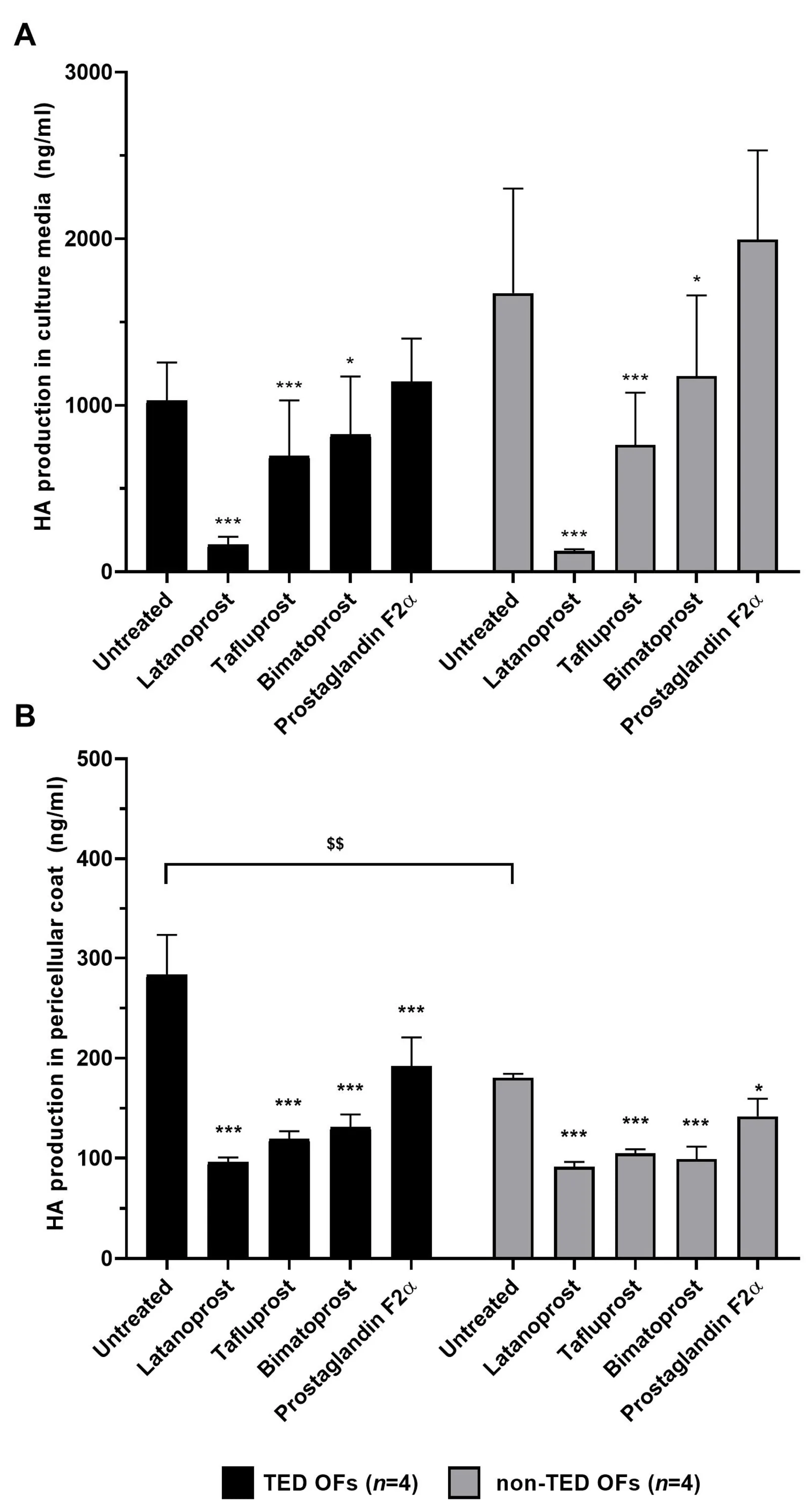
The influence of PGAs and PGF2α (40 µM) on HA production of OFs in (**A**) culture media and in (**B**) pericellular coat. Data were analysed using repeated measures ANOVA followed by Fisher LSD post hoc test. Results are shown as mean ± SEM. *—results compared to untreated, $—TED vs. non-TED comparison; * *p* < 0.05, *** *p* < 0.001, $$ *p* < 0.01.

HA secreted into the culture media and HA present in the pericellular coat of fibroblasts were studied separately. HA production into the culture media by untreated TED and non-TED OFs did not differ (*p* = 0.231). In contrast, the pericellular coat of TED OFs contained a higher amount of HA than that of non-TED OFs (*p* = 0.0010). Each PGA treatment remarkably decreased HA production of OFs, both in the culture media (Figure 2A) and in the pericellular coat (Figure 2B) (*p* < 0.001 and *p* < 0.001, respectively). The magnitude of the decrease did not depend on whether the cells originated from TED or non-TED orbits (*p* = 0.251) (Figure 2).

**Figure 3:**
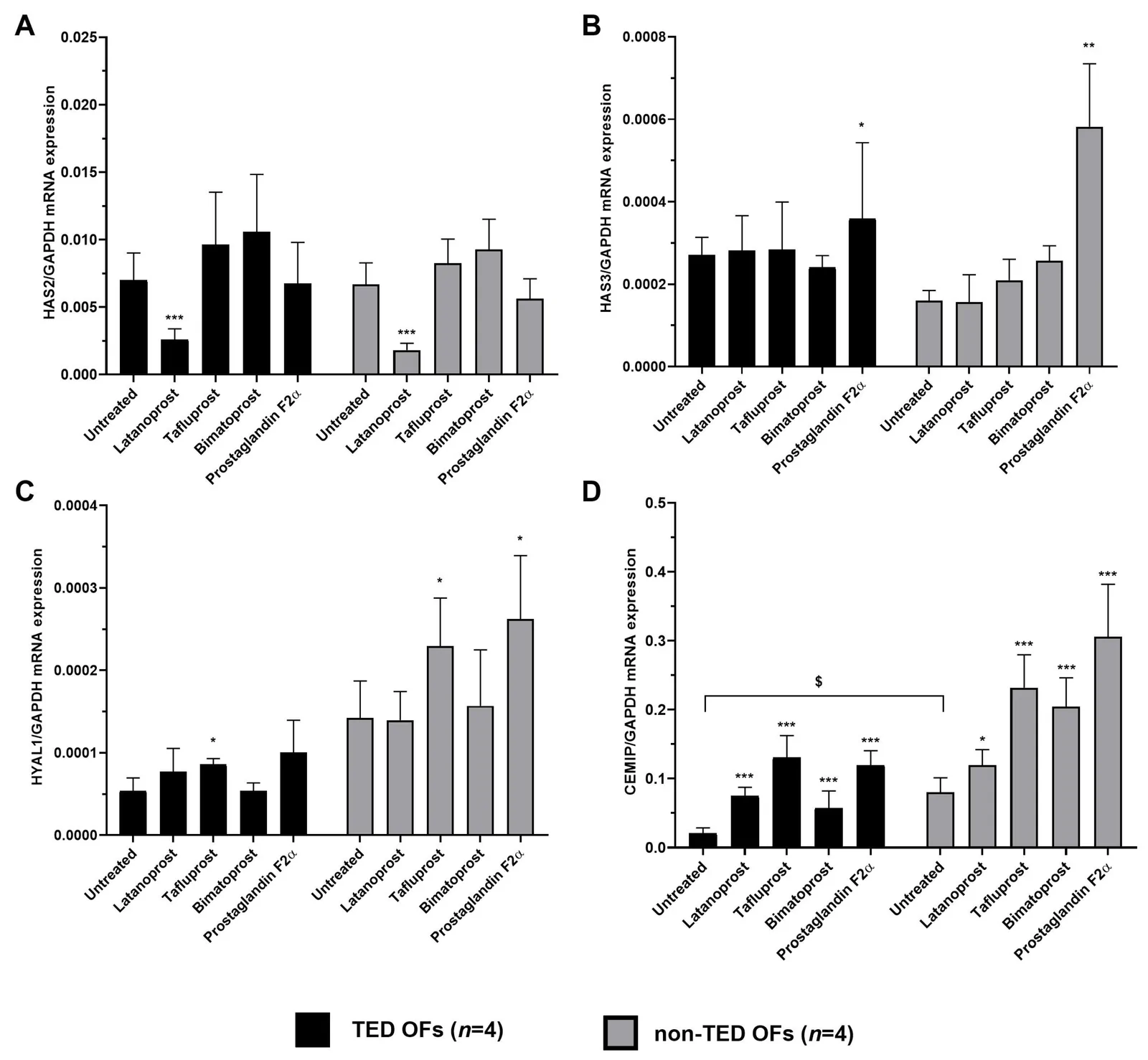
The effect of PGAs and PGF2α on (**A**) *HAS2*, (**B**) *HAS3*, (**C**) *HYAL1* and (**D**) *CEMIP* mRNA expression of TED and non-TED OFs. Data were analysed using repeated measures ANOVA followed by Fisher LSD post hoc test. Results are shown as mean ± SEM. *—compared to untreated, $—TED vs. non-TED comparison; * *p* < 0.05, ** *p* < 0.01, *** *p* < 0.001, $ *p* < 0.05.

**Figure 4:**
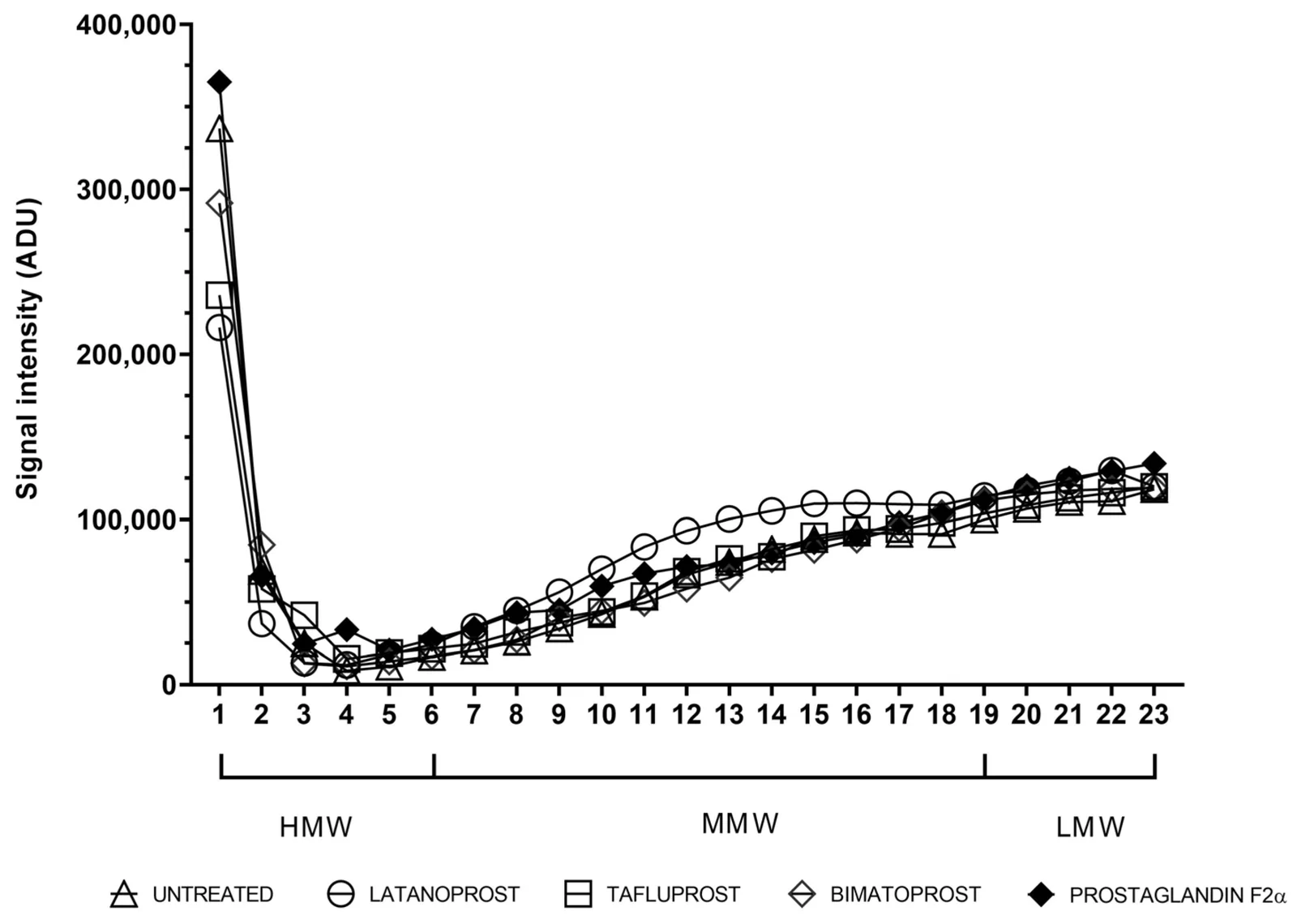
HA molecular weight distribution in the culture media of TED OFs (*n* = 4) after PGAs and PGF2α treatment. Data are expressed as means. ADU: arbitrary densitometry units, HMW—high molecular weight, MMW—medium molecular weight, LMW—low molecular weight.

Subsequently, mRNA levels of enzymes of HA turnover were assessed (Figure 3). Expression levels of *HAS1* and *HYAL2* showed no significant differences in either TED or non-TED OFs following PGA or PGF2α treatment (*p* = 0.419 and *p* = 0.573, respectively). mRNA expression of *HAS2*, *HAS3* and *HYAL1* did not depend on the TED or non-TED origin of the cells (*p* = 0.602, *p* = 0.627 and *p* = 0.214, respectively). Basal (untreated) *CEMIP* mRNA expression was higher in non-TED OFs (*p* = 0.035, Figure 3D). Regarding the effect of individual PGAs, latanoprost was the only PGA that had a reducing effect on *HAS2* expression (*p* < 0.001) (Figure 3A). *HAS3* mRNA expression was slightly increased by PGF2α (*p* = 0.0397) (Figure 3B). Tafluprost increased *HYAL1* expression levels in both TED (*p* = 0.0452) and non-TED OFs (*p* = 0.05), while PGF2α increased *HYAL1* levels only in non-TED OFs (*p* = 0.0403) (Figure 3C). *CEMIP* with its recently discovered hyaluronidase activity had the highest expression levels among examined OF-hyaluronidases. PGAs and PGF2α treatment significantly increased *CEMIP* mRNA expression (please note the different scale of the vertical axis in Figure 3D); according to the post hoc test, each treatment increased *CEMIP* expression levels (*p* < 0.001). Molecular weight distribution of HA present in the culture media in response to PGF2α and each of the PGAs were examined. Besides decreasing HA production in culture media, latanoprost also modified the distribution of HA: HMW-HA decreased both in TED and non-TED OFs (*p* < 0.001 and *p* = 0.0046, respectively), while MMW-HA increased only in TED OFs (*p* < 0.001). A clear shift was observed in the molecular weight distribution of HA, from HMW-HA towards MMW-HA, in TED OFs following latanoprost treatment (Figure 4).

**Figure 5:**
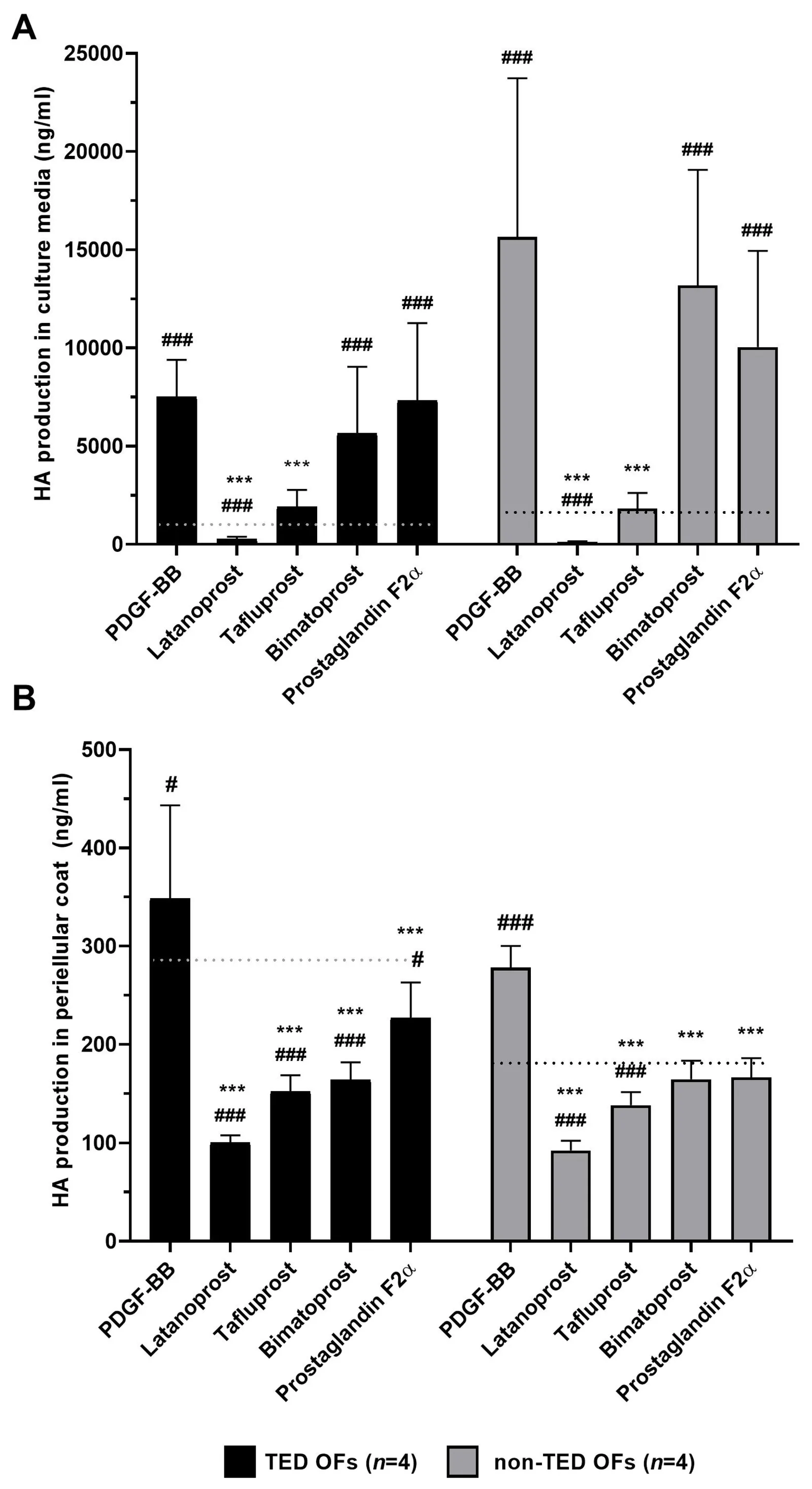
The influence of PGAs and PGF2α (40 µM) on PDGF-BB-stimulated HA production of OFs in the culture media (**A**) and in the pericellular coat (**B**). Data were analysed using repeated measures ANOVA followed by Fisher LSD post hoc test. Results are shown as mean ± SEM. The dotted lines represent the mean HA production of untreated cultures. #—results compared to untreated, *—results compared to PDGF-BB-stimulated; # *p* < 0.05, ### *p* < 0.001, *** *p* < 0.001.

Regardless of their site of origin (*p* = 0.187), cells responded uniformly to PDGF-BB treatment with increased HA production both in culture media (*p* < 0.001) and in the pericellular coat (*p* < 0.001) (Figure 5).

In the culture media, latanoprost not only diminished the stimulatory effect of PDGF-BB (*p* < 0.001), but was also able to reduce it below untreated levels (*p* < 0.001) (Figure 5A) in both TED and non-TED OFs. Tafluprost reduced the PDGF-BB induced HA levels (*p* < 0.001) back to basal levels (*p* = 0.474). Bimatoprost and PGF2α were unable to counteract the HA-increasing effect of PDGF-BB.

**Figure 6:**
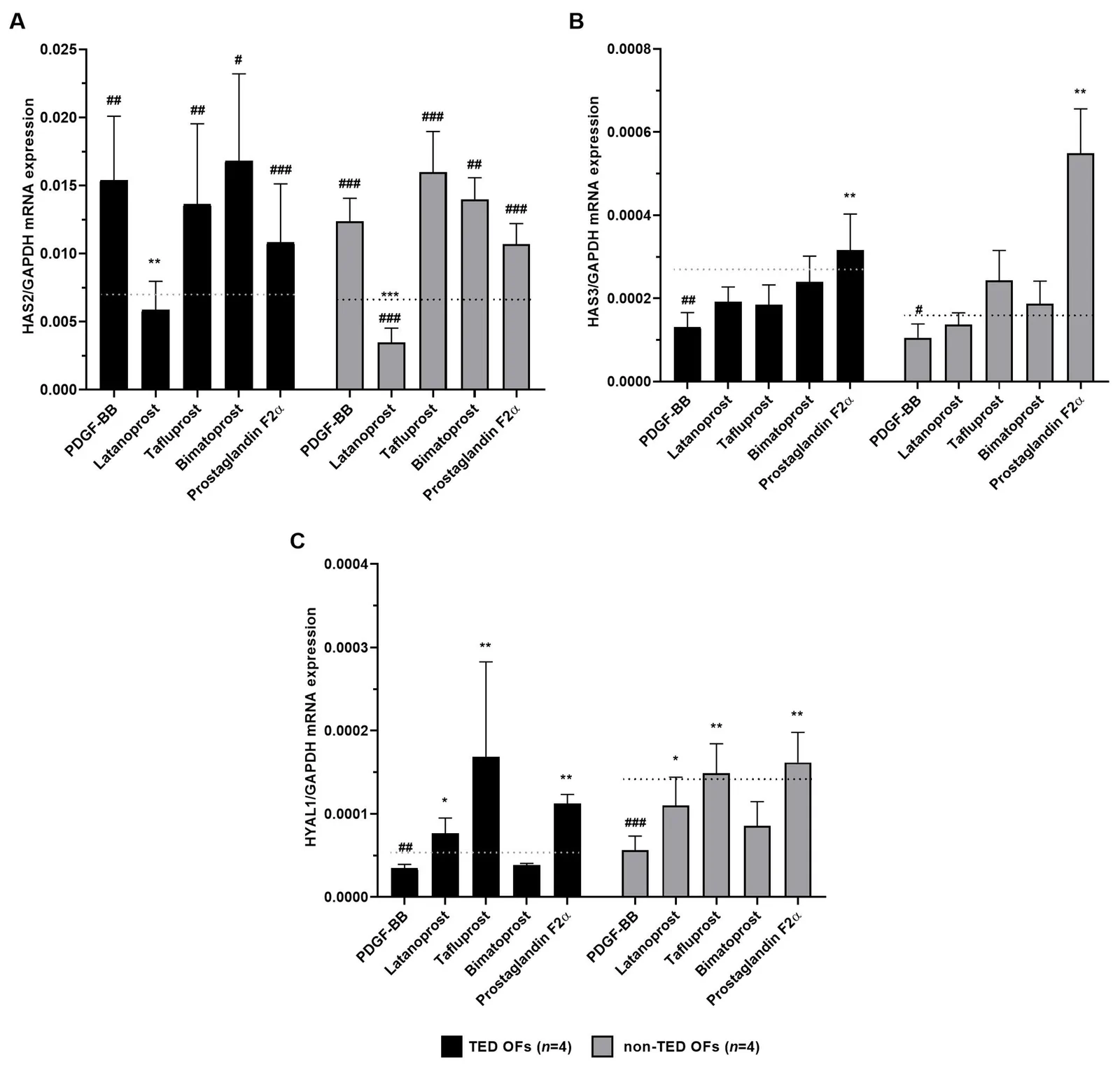
The effect of PGAs and PGF2α (40 µM) on PDGF-BB (10 ng/mL) induced (**A**) *HAS2*, (**B**) *HAS3* and (**C**) *HYAL1* mRNA expression. Data were analysed using repeated measures ANOVA followed by Fisher LSD post hoc test. Results are shown as mean ± SEM. The dotted lines represent the mean mRNA expressions of untreated cultures. #—results compared to untreated, *—results compared to PDGF-BB-stimulated; # *p* < 0.05, ## *p* < 0.01, ### *p* < 0.001, * *p* < 0.05, ** *p* < 0.01, *** *p* < 0.001.

In the pericellular coat, each PGF2α and PGAs resulted in a constant reduction in HA content (*p* < 0.001), counteracting PDGF-BB stimulation, albeit to different degrees (Figure 5B). In TED OFs, all treatment types were effective not only in diminishing PDGF-BB-induced HA production (*p* < 0.001) but also in reducing the pericellular HA below the initial unstimulated level (*p* < 0.001). Non-TED OFs behaved similarly in the presence of latanoprost and tafluprost, while the suppressive effects of bimatoprost and PGF2α were less marked. PGAs and PGF2α did not affect *HAS1* and *HYAL2* mRNA expression, either in TED or in non-TED OFs, with or without PDGF-BB treatment (*p* = 0.252 and *p* = 0.597, respectively). While PGF2α and each PGA increased *CEMIP* expression, PDGF-BB did not show such an effect (*p* = 0.291) *HAS2* mRNA expression increased in response to PDGF-BB both in TED and non-TED OFs (*p* = 0.0088 and *p* ≤ 0.001, respectively) (Figure 6A). PDGF-BB-stimulated *HAS2* expression reached the untreated level in response to latanoprost; this is consonant with the finding that latanoprost was the only PGA to be able to effectively decrease *HAS2* expression in unstimulated cells (Figure 3A, above). *HAS3* expression was inhibited by PDGF-BB treatment (*p* = 0.0030) regardless of the origin of the cells (*p* = 0.258) (Figure 6B). PGF2α uniquely upregulated *HAS3* expression (*p* = 0.0019). *HYAL1* mRNA level was decreased by PDGF-BB (*p* < 0.001) regardless of the origin of the cells (*p* = 0.214); latanoprost, tafluprost and PGF2α were able to antagonise this to different extents (*p* = 0.0061, *p* = 0.0092, and *p* < 0.001, respectively) (Figure 6C).

**Figure 7:**
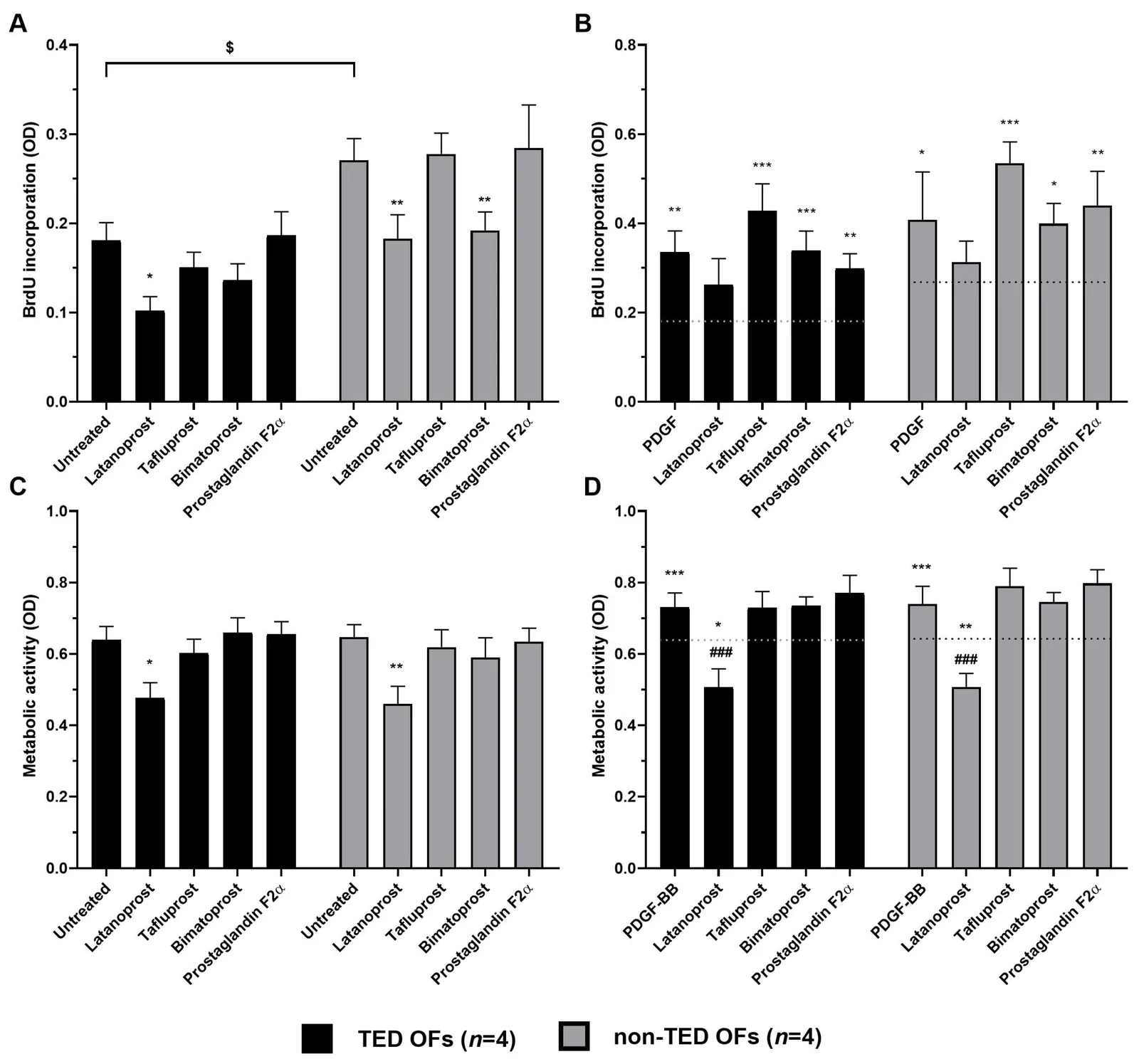
The proliferation potential and metabolic activity of OFs. (**A**) Proliferation in the presence of each PGA or PGF2α alone, or (**B**) in combination with PDGF-BB (**B**). Metabolic activity following PGA or PGF2α treatment without (**C**) and with (**D**) PDGF-BB stimulation. Data were analysed using repeated measures ANOVA followed by Fisher LSD post hoc test. Results are shown as mean ± SEM. The dotted lines represent the BrdU incorporation (**B**) or metabolic activity (**D**) of untreated cultures. #—results compared to untreated, *—results compared to PDGF-BB-stimulated, $—TED vs. non-TED comparison; ### *p* < 0.001, * *p* < 0.05, ** *p* < 0.01, *** *p* < 0.001, $ *p* < 0.05.

Proliferation potential, estimated by BrdU incorporation, of non-TED OFs was higher than TED OFs’ (*p* = 0.0295) (Figure 7A); this difference disappeared after PDGF-BB stimulation, when proliferation slightly increased in both TED and non-TED OFs (*p* < 0.001 and *p* = 0.0197, respectively). Latanoprost decreased the proliferation rate of both TED OFs (*p* = 0.05) and non-TED OFs (*p* = 0.0067). Following PDGF-BB stimulation, latanoprost was also effective in this respect (Figure 7B).

**Figure 8:**
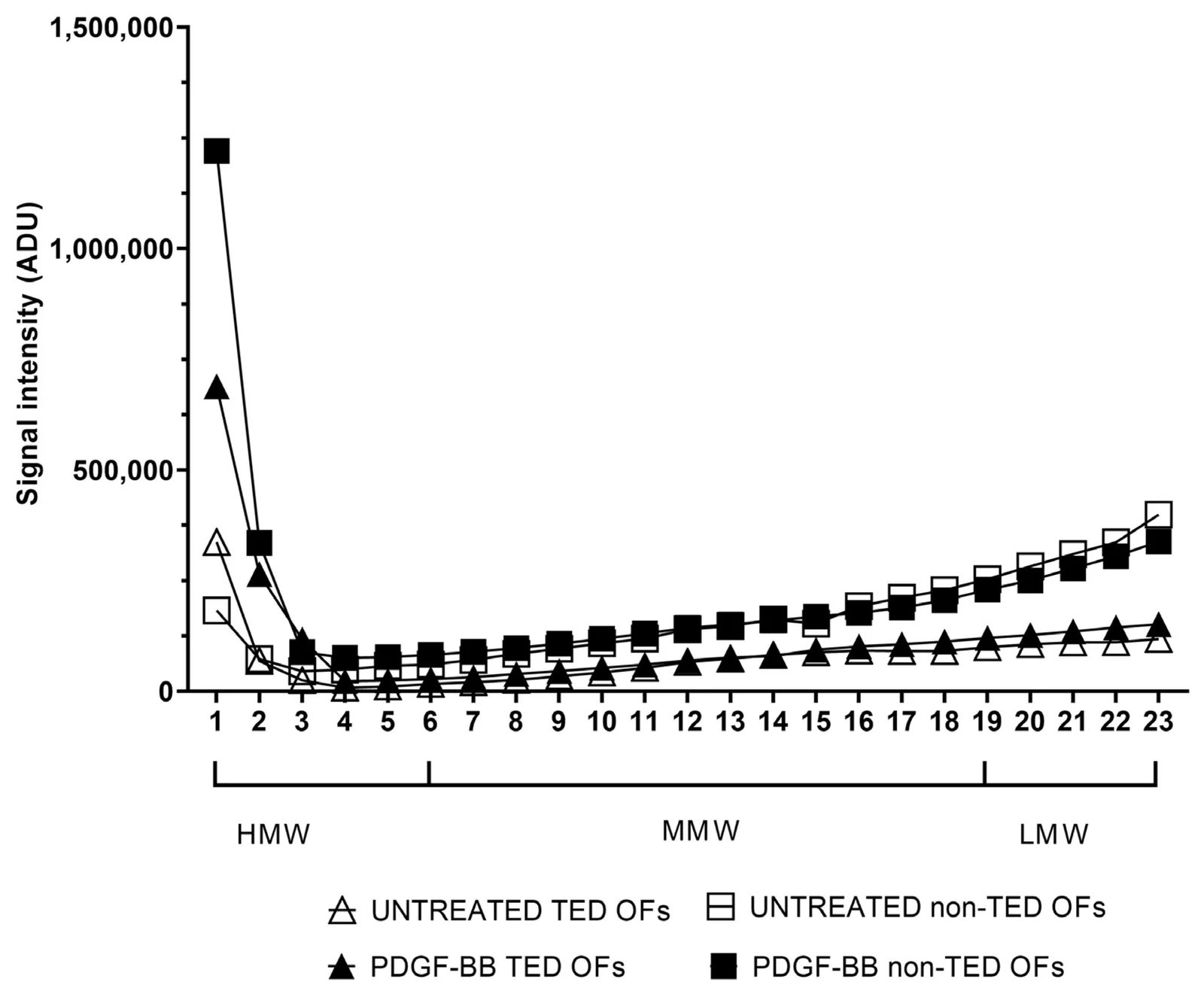
The change in HA molecular weight distribution after PDGF-BB treatment in the culture media of TED OFs (*n* = 4) and non-TED OFs (*n* = 4). Data are expressed as means. ADU: arbitrary densitometry units, HMW—high molecular weight, MMW—medium molecular weight, LMW—low molecular weight.

The pattern of behaviour of OFs in the metabolic activity measurements resembled what had been seen in proliferation; cells’ metabolic activity was only reduced by latanoprost (TED OFs *p* = 0.0202 and non-TED OFs *p* = 0.0086) (Figure 7C). Again, PDGF-BB stimulation slightly but significantly increased the metabolic activity in OFs (*p* < 0.001); latanoprost was the only PGA able to reduce it below baseline level (TED OFs *p* = 0.0295 and non-TED OFs *p* = 0.006) (Figure 7D). Agarose gel electrophoresis revealed that increased HA production into the culture media after PDGF-BB stimulation was caused exclusively by an increase in HMW-HA (Figure 8).

**Figure 9:**
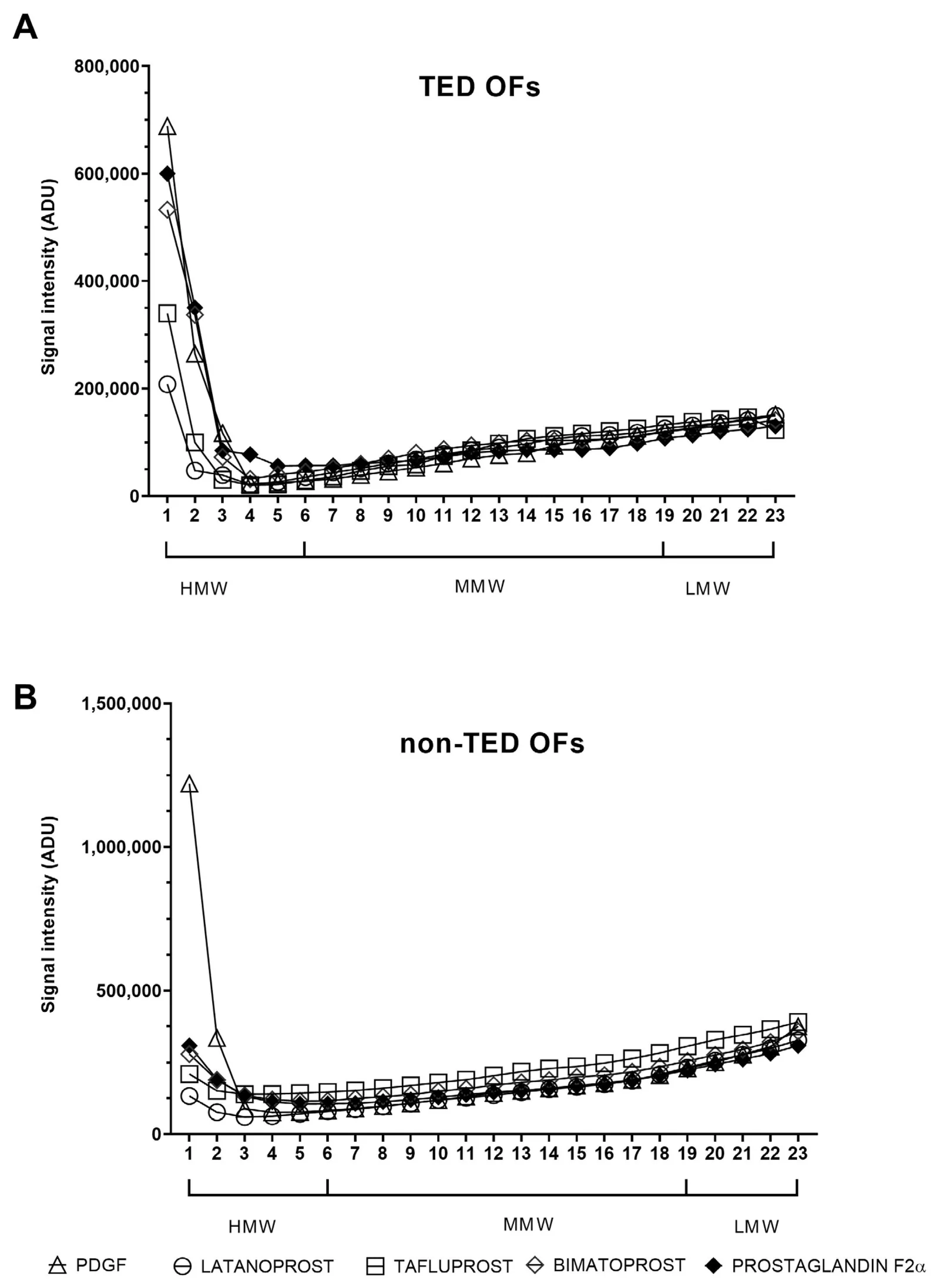
HA molecular weight distribution in the culture media of (**A**) TED (*n* = 4) and (**B**) non-TED (*n* = 4) OFs treated with PDGF-BB alone or together with a PGA or PGF2α. Data are expressed as means. ADU: arbitrary densitometry units. HMW—high molecular weight, MMW—medium molecular weight, LMW—low molecular weight.

Each studied PGA decreased HMW-HA production in both cell types (Figure 9). The reduction in the quantity of HMW-HA was more marked in non-TED OFs compared to TED OFs; latanoprost was the most effective PGA in this respect.

## 4. Discussion

TED involves the swelling of soft tissues within the constraints of the bony orbit, mainly caused by excess HA accumulation, increased fibroblast proliferation, and fat cell development [31]. High water-binding capacity of HA contributes to tissue oedema exacerbating orbital congestion [32,33]. Pro-inflammatory and pro-fibrotic factors, such as interleukins, transforming growth factor beta (TGF-β) and PDGF-BB are elevated during the active phase, promoting ECM accumulation and orbital tissue remodelling [34]. Elevated IOP in TED primarily results from congestion of expanded orbital tissues and impaired aqueous humour outflow via the uveoscleral pathway [35]. IOP-lowering medications are commonly used as part of the management strategy [31]. PGAs are among the first-line treatments for glaucoma. However, their effects go beyond lowering IOP. A notable side effect, DUES, has been linked to orbital fat atrophy, which is likely mediated by inhibition of adipogenesis and downregulation of PPARγ in preadipocytes [25].

Numerous data support that the PGAs have a role in the remodelling of the ECM. Studies indicate that PGF2α enhances collagen production in cardiac fibroblasts, promoting fibrosis [21], whereas research on prostaglandin E2 (PGE_2_) in dermal fibroblasts has shown inhibition of TGF-β_1_-induced collagen synthesis and prevention of hypertrophic scarring [22]. Latanoprost increases the matrix metalloproteinases (MMP) -3 and -17 and induces the expression of MMP-9 [36]. Tissue inhibitor of metalloproteinase (TIMP) -3 is known to be upregulated to compensate MMPs in human ciliary body tissue and explant cultures of ciliary body smooth muscle cells [36]. MMPs have been found upregulated and fibronectin downregulated by bimatoprost, which also has a role in the remodelling of the ECM. These together may promote sustained outflow and tissue remodelling [37]. Latanoprost selectively upregulated MMP-3 and TIMP-2 in tenon fibroblasts [38]. PGAs and PGF2α modulated ECM remodelling during adipogenesis in 3D human OF organoids, providing insight into the possible molecular mechanisms underlying DUES [39]. Bimatoprost has been shown to decrease the size of 3D organoids of OFs of TED patients and downregulate key ECM genes while upregulating interleukin-6, indicating a direct modulatory effect on OFs’ structure and inflammatory response [40]. PGE2 elicits characteristic morphological changes in OFs from TED patients, whereas dermal fibroblasts remain unaffected. This phenomenon occurs via the prostaglandin E2 receptor 2 and cAMP signalling, supporting the idea that prostaglandins may be important mediators in the inflammatory and remodelling processes of orbital connective tissue [41].

Although recent studies have explored prostaglandin effects on fibroblasts [42,43], to the best of our knowledge, their impact on HA production in OFs in the context of TED has not been explored. We aimed to investigate the effects of latanoprost, tafluprost, bimatoprost, and prostaglandin F_2_α on HA production of OFs. Despite the rapid systemic degradation of the eye drop’s active substance, its ocular hypotensive effect is long-lasting and is maintained for at least 24 h [44,45,46]. The HA modulating effect of the PGAs was investigated at 72 h. The present study used a 40 µM experimental concentration of PGAs, which exceeds the physiological levels recorded in the anterior chamber after routine clinical application. Standard pharmacokinetic data in humans indicate that a single topical dose of 0.005% latanoprost generates a peak concentration of only 10^−7^ M in the aqueous humour [45]. These PGAs reduced HA in the OF’s culture media; latanoprost and tafluprost were the most effective. Each tested PGA and PGF_2_α also reduced HA in the pericellular coat. Each compound modulated mRNA expression of HA-homeostasis enzymes: latanoprost selectively reduced *HAS2*, PGF2α mildly upregulated *HAS3*, tafluprost increased *HYAL1* in both TED and non-TED OFs, and PGF2α in non-TED OFs only. In the current study, each PGA and PGF2α upregulated mRNA expression of *CEMIP*. These findings suggest a dual mechanism that leads to decreased HA accumulation in the presence of a PGA or PGF2α: suppression of synthesis via selective HAS downregulation, and potentially enhanced degradation primarily through *CEMIP* induction. Furthermore, we cannot rule out that the significant decrease in metabolic activity induced by latanoprost treatment partially contributes to the lower absolute HA levels and the decline in gene expression. This trend could, in part, reflect a reduced number of metabolically active cells at the end of the incubation period rather than a purely isolated effect on HA turnover. This observation aligns with our previous findings with 4-methylumbelliferone, where a robust reduction in HA production was accompanied by suppressed metabolic activity without a direct impact on overall cell viability [47]. The different enzymatic responses of TED and non-TED OFs highlight the complex regulation of HA metabolism. Modulation of CEMIP may represent a key pathway in controlling HA turnover.

PDGF-BB stimulation mimics inflammatory activation and increases HA production both in the culture media and in the pericellular coat of OFs [48]. Most effectively, latanoprost counteracted this induction in the culture media, followed by tafluprost, while bimatoprost and PGF2α failed to reverse it. In the pericellular coat, all treatments reduced HA after PDGF-BB stimulation; in TED OFs it dropped below baseline, while in non-TED OFs latanoprost and tafluprost were again the most effective, while bimatoprost and PGF2α only partially mitigated the increase. Latanoprost and tafluprost modulate both basal and PDGF-BB-induced HA accumulation, suggesting a mechanism by which PGAs may influence orbital ECM remodelling. The observed reduction in pericellular HA content following PGA exposure may suggest a potential effect on extracellular matrix hydration; however, whether this effect translates into reduced oedema or orbital tissue swelling in vivo remains to be determined. While our 40 µM model served as an essential in vitro tool to definitively characterise the results of PGA treatment on HA turnover, caution must be exercised when translating these findings directly to in vivo clinical settings. Further studies using in vitro cell and tissue models are needed to elucidate the precise molecular pathways by which PGAs exert their effects on deeper orbital tissues, and their long-term impact on orbital tissue architecture. To address these questions, an animal model could provide a suitable approach.

The primary limitation of this study is that our PDGF-BB-induced in vitro model cannot account for the complex interplay of factors within the local orbital environment, as it focuses on a single component of the inflammatory process. A potential limitation of our findings is that their translational value is hard to estimate. Due to their lipophilic molecular characteristics, PGAs are assumed to reach deep orbital tissues when used in eyedrops [48,49]; however, up till now, no direct evidence of this has been reported. It should be noted that the concentrations tested here were not aimed to be equivalent to once-daily topical treatments; therefore, further pharmacokinetic studies are required to determine whether these effects occur at physiological in vivo concentrations [50]. The clinical background of the orbital tissues used in our study may be a limitation that could influence the phenotype of the established cell cultures and limit the generalizability of our results. TED OFs are derived from patients previously treated with intravenous glucocorticoids or orbital radiotherapy, which may permanently alter the characteristics of the cells. Finally, due to the scarcity of these surgical tissues, our study was constrained to a small sample size. Furthermore, the use of Fisher’s LSD test for post hoc analysis carries an increased risk of Type I error, and marginally significant findings must be interpreted with caution, even though post hoc analysis was only performed when the ANOVA indicated a significant difference. These practical limitations inherent in rare human primary samples should be carefully considered when extrapolating our in vitro results to broader clinical populations.

In summary, PGAs attenuated HA accumulation in orbital fibroblast cultures under both basal and PDGF-BB-stimulated conditions, suggesting that these agents may modulate HA metabolism in OFs. Although the present findings are confined to an in vitro model and do not permit direct conclusions regarding orbital oedema, disease progression, or therapeutic efficacy in TED, they may be of potential clinical interest given the established use of PGAs in glaucoma management, including secondary glaucoma in patients with TED. Further studies are warranted to determine whether these cellular effects translate into meaningful changes in orbital tissue biology in vivo.

## Data Availability

The data presented in this study are available on request from the corresponding author due to restrictions related to patient privacy and ethical considerations.

## Abbreviations

The following abbreviations are used in this manuscript:

BrdU	5-Bromo-2′-deoxyuridine
cDNA	Complementary DNA
CEMIP	Cell Migration-Inducing and Hyaluronan-Binding Protein
DMSO	Dimethyl Sulfoxide
DMEM	Dulbecco’s Modified Eagle Medium
DPBS	Dulbecco’s Phosphate-Buffered Saline
DUES	Deepening of the Upper Eyelid Sulcus
ECM	Extracellular Matrix
ELISA	Enzyme-Linked Immunosorbent Assay
FBS	Foetal Bovine Serum
HA	Hyaluronan
HAS	Hyaluronan Synthases
HMW	High Molecular Weight
HYAL	Hyaluronan-Degrading Enzymes
IOP	Intraocular Pressure
LMW	Low Molecular Weight
MMW	Medium Molecular Weight
MTT	3-(4,5-Dimethylthiazol-2-yl)-2,5-Diphenyltetrazolium Bromide
OAG	Open-Angle Glaucoma
OHT	Ocular Hypertension
OFs	Orbital Fibroblasts
PDGF-BB	Platelet-Derived Growth Factor-BB
PGAs	Prostaglandin Analogues
PGF2α	Prostaglandin F2α
SDS	Sodium Dodecyl Sulfate
SG	Stable Glutamine
TBE	Tris–Borate–EDTA
TED	Thyroid Eye Disease

## References

[1] Smith T.J., Hegedüs L. (2016). Graves’ Disease. N. Engl. J. Med..

[2] Lin H., Duan H., Zheng J., Jiang Z., Xu Y., Huang H. (2025). Clinical Characteristics of Thyroid Eye Disease and Expression Profile of Peripheral Blood Immune Cells. Sci. Rep..

[3] Zhang X., Zhao Q., Li B. (2023). Current and Promising Therapies Based on the Pathogenesis of Graves’ Ophthalmopathy. Front. Pharmacol..

[4] Hou T.-Y., Wu S.-B., Kau H.-C., Tsai C.-C. (2021). The Role of Oxidative Stress and Therapeutic Potential of Antioxidants in Graves’ Ophthalmopathy. Biomedicines.

[5] Smith T.J., Janssen J.A.M.J.L. (2019). Insulin-like Growth Factor-I Receptor and Thyroid-Associated Ophthalmopathy. Endocr. Rev..

[6] Hargittai I., Magdolna H. (2008). Molecular Structure of Hyaluronan: An Introduction. Struct. Chem..

[7] Waldenstrm A., Martinussen H.J., Gerdin B., Hallgren R. (1991). Accumulation of Hyaluronan and Tissue Edema in Experimental Myocardial Infarction. J. Clin. Investig..

[8] Itano N., Kimata K. (2002). Mammalian Hyaluronan Synthases. IUBMB Life.

[9] Hascall V.C., Wang A., Tammi M., Oikari S., Tammi R., Passi A., Vigetti D., Hanson R.W., Hart G.W. (2015). The Dynamic Metabolism of Hyaluronan Regulates the Cytosolic Concentration of UDP-GlcNAc. Matrix Biol..

[10] Yoshida H., Nagaoka A., Kusaka-kikushima A., Tobiishi M., Kawabata K., Sayo T., Sakaia S., Sugiyama Y., Enomotob H., Okadac Y. (2013). KIAA1199, a Deafness Gene of Unknown Function, Is a New Hyaluronan Binding Protein Involved in Hyaluronan Depolymerization. Proc. Natl. Acad. Sci. USA.

[11] Kobayashi T., Chanmee T., Itano N. (2020). Hyaluronan: Metabolism and Function. Biomolecules.

[12] Di Meo C., Stellavato A., D’Agostino M., D’Agostino A., Schiraldi C., La Gatta A. (2024). Hyaluronan Size and Concentration: Effect on Key Biophysical and Biochemical Features. Int. J. Biol. Macromol..

[13] Wang X., Liu X., Li C., Li J., Qiu M., Wang Y., Han W. (2025). Effects of Molecular Weights on the Bioactivity of Hyaluronic Acid: A Review. Carbohydr. Res..

[14] Monslow J., Govindaraju P., Puré E. (2015). Hyaluronan—A Functional and Structural Sweet Spot in the Tissue Microenvironment. Front. Immunol..

[15] Karhanová M., Kalitová J., Kovář R., Schovánek J., Karásek D., Čivrný J., Hübnerová P., Mlčák P., Šín M. (2022). Ocular Hypertension in Patients with Active Thyroid-Associated Orbitopathy: A Predictor of Disease Severity, Particularly of Extraocular Muscle Enlargement. Graefe’s Arch. Clin. Exp. Ophthalmol..

[16] Kim J.W., Ko J., Woo Y.J., Bae H.W., Yoon J.S., Hospital S., Bom Y., Clinic E., Yoon J.S. (2018). Prevalence of Ocular Hypertension and Glaucoma as Well as Associated Factors in Graves’ Orbitopathy. J. Glaucoma.

[17] Orbitopathy I., Behrouzi Z., Rabei H.M., Azizi F., Daftarian N., Mehrabi Y., Ardeshiri M., Mohammadpour M. (2007). Prevalence of Open-Angle Glaucoma, Glaucoma Suspect, and Ocular Hypertension in Thyroid-Related Immune Orbitopathy. J. Glaucoma.

[18] Dams I., Wasyluk J., Prost M., Kutner A. (2013). Therapeutic Uses of Prostaglandin F2α Analogues in Ocular Disease and Novel Synthetic Strategies. Prostaglandins Other Lipid Mediat..

[19] Tappeiner C., Perren B., Iliev M.E., Frueh B.E., Goldblum D. (2008). Orbitale Fettgewebsatrophie Bei Lokaler Bimatoprost-Therapie - Kann Bimatoprost Einen Enophthalmus Verursachen?. Klin. Monbl. Augenheilkd..

[20] Holló G. (2007). The Side Effects of the Prostaglandin Analogues. Expert Opin. Drug Saf..

[21] Ding W., Ti Y., Wang J., Wang Z., Xie G., Shang Y., Tang M., Zhang Y., Zhang W., Zhong M. (2012). Prostaglandin F2α Facilitates Collagen Synthesis in Cardiac Fibroblasts via an F-Prostanoid Receptor/Protein Kinase C/Rho Kinase Pathway Independent of Transforming Growth Factor Β1. Int. J. Biochem. Cell Biol..

[22] Zhao J., Shu B., Chen L., Tang J., Zhang L., Xie J., Liu X., Xu Y., Qi S. (2016). Prostaglandin E2 Inhibits Collagen Synthesis in Dermal Fibroblasts and Prevents Hypertrophic Scar Formation in Vivo. Exp. Dermatol..

[23] Hata A.N., Breyer R.M. (2004). Pharmacology and Signaling of Prostaglandin Receptors: Multiple Roles in Inflammation and Immune Modulation. Pharmacol. Ther..

[24] Taketani Y., Yamagishi R., Fujishiro T., Igarashi M., Sakata R., Aihara M. (2014). Activation of the Prostanoid FP Receptor Inhibits Adipogenesis Leading to Deepening of the Upper Eyelid Sulcus in Prostaglandin-Associated Periorbitopathy. Investig. Ophthalmol. Vis. Sci..

[25] Choi H.Y., Lee J.E., Lee J.W., Park H.J., Jung J.H. (2012). In Vitro Study of Antiadipogenic Profile of Latanoprost, Travoprost, Bimatoprost, and Tafluprost in Human Orbital Preadiopocytes. J. Ocul. Pharmacol. Ther..

[26] Kahaly G.J., Bartalena L., Hegedüs L., Leenhardt L., Poppe K., Pearce S.H. (2018). 2018 European Thyroid Association Guideline for the Management of Graves’ Hyperthyroidism. Eur. Thyroid J..

[27] Bahn R.S., Gorman C.A., Woloschak G.E., David C.S., Johnson P.M., Johnson C.M. (1987). Human Retroocular Fibroblasts in Vitro: A Model for the Study of Graves’ Ophthalmopathy. J. Clin. Endocrinol. Metab..

[28] Hyttinenj M., Tammir H., Tammim I. (2014). Hyaluronan Synthase 1 (HAS1) Produces a Cytokine-and Glucose-Inducible, CD44-Dependent Cell Surface Coat. Exp. Cell Res..

[29] Galgoczi E., Jeney F., Gazdag A., Erdei A., Katko M., Nagy D.M., Ujhelyi B., Steiber Z., Gyory F., Berta E. (2016). Cell Density-Dependent Stimulation of PAI-1 and Hyaluronan Synthesis by TGF-β in Orbital Fibroblasts. J. Endocrinol..

[30] Galgoczi E., Katko M., Borbely S., Orsos I., Molnar Z., Ujhelyi B., Steiber Z., Nagy E.V. (2025). Agarose Gel Electrophoresis Reveals the Molecular Weight Distribution of Hyaluronan Produced by Orbital Fibroblasts. Gels.

[31] Sevimli N., Eryılmaz M., Azarbaz M.K., Taydaş G. (2025). The Impact of Graves’ Orbitopathy on Anterior Segment Structures and Choroidal Parameters. BMC Ophthalmol..

[32] Galgoczi E., Molnar Z., Katko M., Ujhelyi B., Steiber Z., Nagy E.V. (2024). Cyclosporin A Inhibits PDGF-BB Induced Hyaluronan Synthesis in Orbital Fibroblasts. Chem. Biol. Interact..

[33] Draman M.S., Zhang L., Dayan C., Ludgate M. (2021). Orbital Signaling in Graves’ Orbitopathy. Front. Endocrinol..

[34] Chiu H., Wu S., Tsai C.-C. (2024). The Role of Fibrogenesis and Extracellular Matrix Proteins in the Pathogenesis of Graves’ Ophthalmopathy. Int. J. Mol. Sci..

[35] Nassr M.A., Morris C.L., Netland P.A., Karcioglu Z.A. (2009). Intraocular Pressure Change in Orbital Disease. Surv. Ophthalmol..

[36] Oh D., Martin J.L., Williams A.J., Peck R.E., Pokorny C., Russell P., Birk D.E., Rhee D.J. (2006). Analysis of Expression of Matrix Metalloproteinases and Tissue Inhibitors of Metalloproteinases in Human Ciliary Body after Latanoprost AND. Investig. Ophthalmol. Vis. Sci..

[37] Stamer W.D., Perkumas K.M., Kang M.H., Dibas M., Robinson M.R., Rhee D.J. (2023). Proposed Mechanism of Long-Term Intraocular Pressure Lowering With the Bimatoprost Implant. Investig. Ophthalmol. Vis. Sci..

[38] Mietz H., Esser J.M., Welsandt G., Kociok N., Hueber A., Joussen A., Esser P., Krieglstein G.K. (2003). Latanoprost Stimulates Secretion of Matrix Metalloproteinases in Tenon Fibroblasts Both In Vitro and In Vivo. Investig. Ophthalmol. Vis. Sci..

[39] Itoh K., Hikage F., Ida Y., Ohguro H. (2020). Prostaglandin F2α Agonists Negatively Modulate the Size of 3D Organoids from Primary Human Orbital Fibroblasts. Investig. Ophthalmol. Vis. Sci..

[40] Ichioka H., Ida Y., Watanabe M., Ohguro H., Hikage F. (2021). Prostaglandin F2α and EP2 Agonists, and a ROCK Inhibitor Modulate the Formation of 3D Organoids of Grave’s Orbitopathy Related Human Orbital Fibroblasts. Exp. Eye Res..

[41] Wang H.S., Keese C.R., Giaever I., Smith T.J. (1995). Prostaglandin E2 Alters Human Orbital Fibroblast Shape through a Mechanism Involving the Generation of Cyclic Adenosine Monophosphate. J. Clin. Endocrinol. Metab..

[42] Zhu R., Wang X., Wang B., Ouyang X., You Y., Xie H., Zhang M.-C., Jiang F.-G. (2023). Prostaglandin F2 α Regulates Adipogenesis by Modulating Extracellular Signal-Regulated Kinase Signaling in Graves’ Ophthalmopathy. Int. J. Mol. Sci..

[43] Li K., Zhao J., Wang M., Niu L., Wang Y., Li Y., Zheng Y. (2021). The Roles of Various Prostaglandins in Fibrosis: A Review. Biomolecules.

[44] Perry C.M., Mcgavin J.K., Culy C.R., Ibbotson T. (2003). Latanoprost: An Update of Its Use in Glaucoma and Ocular Hypertension. Drugs Aging.

[45] Sjöquist B., Stjernschantz J. (2002). Ocular and Systemic Pharmacokinetics Of Latanoprost in Humans. Surv. Ophthalmol..

[46] Society E.G. (2025). Terminology and Guidelines for Glaucoma, 6th Edition. Br. J. Ophthalmol..

[47] Galgoczi E., Jeney F., Katko M., Erdei A., Gazdag A., Sira L., Bodor M., Berta E., Ujhelyi B., Steiber Z. (2020). Characteristics of Hyaluronan Synthesis Inhibition by 4-Methylumbelliferone in Orbital Fibroblasts. Investig. Ophthalmol. Vis. Sci..

[48] van Steensel L., Hooijkaas H., Paridaens D., den Bosch W.A.v., Kuijpers R.W.A.M., Drexhage H.A., van Hagen P.M., Dik W.A. (2015). PDGF Enhances Orbital Fibroblast Responses to TSHR Stimulating Autoantibodies in Graves’ Ophthalmopathy Patients. J. Clin. Endocrinol. Metab..

[49] Chen J.Y., Le A., Caprioli J., Giaconi J.A., Nouri-mahdavi K., Law S.K., Bonelli L., Coleman A.L., Demer J.L. (2020). Orbital Fat Volume After Treatment with Topical Prostaglandin Agonists. Investig. Ophthalmol. Vis. Sci..

[50] Madhu C., Rix P., Nguyen T., Chien D., Woodward D.F., Tang-liu D.D.S. (1998). Penetration of Natural Prostaglandins and Their Ester Prodrugs and Analogs Across Human Ocular Tissues in Vitro. J. Ocul. Pharmacol. Ther..

